# Sleep and Health-Related Characteristics among Adolescents during COVID-19: An Update

**DOI:** 10.3390/ijerph19095078

**Published:** 2022-04-21

**Authors:** Joëlle N. Albrecht, Helene Werner, Noa Rieger, Oskar G. Jenni, Reto Huber

**Affiliations:** 1 Child Development Center, University Children’s Hospital Zurich, University of Zurich (UZH), 8032 Zurich, Switzerland; joelle.albrecht@hotmail.com (J.N.A.); helene.werner@kispi.uzh.ch (H.W.); noa.rieger@gmail.com (N.R.); oskar.jenni@kispi.uzh.ch (O.G.J.); 2 Children’s Research Center, University Children’s Hospital Zurich, University of Zurich (UZH), 8032 Zurich, Switzerland; 3 Department of Psychosomatics and Psychiatry, University Children’s Hospital Zurich, University of Zurich (UZH), 8032 Zurich, Switzerland; 4 Department of Child and Adolescent Psychiatry and Psychotherapy, University Hospital of Psychiatry, University of Zurich (UZH), 8032 Zurich, Switzerland

**Keywords:** pandemic lockdown, high school closures, sleep duration, school start times, adolescents’ health

## Abstract

Two opposing effects on adolescents’ health during COVID-19 lockdown have been described: A beneficial one due to longer sleep times during school closures and a detrimental one of psychological distress. This study investigated how sleep and health changed in the course of the pandemic when schools were open again. Overall, 12,238 adolescents in Switzerland participated in three cross-sectional online surveys: In 2017 under regular conditions (control group), during pandemic school closures in 2020 (closure group), and in 2021 still under pandemic conditions, but schools were open again (postclosure group). Sleep behavior and health-related characteristics (health-related quality of life; caffeine, alcohol, and nicotine use) in all three groups and depressive symptoms in the closure and postclosure groups were assessed. The sleep period on school days was longer in the closure group (median 9.00 h, interquartile range 8.25–9.75) and similar in the postclosure (7.92, 7.00–8.50) compared to the control group (7.75, 7.08–8.33). Health-related characteristics were better during school closures and similar to worse in the postclosure compared to the control group. Depressive symptom levels were higher in the postclosure than in the closure group. Therefore, beneficial effects were specific to school closures and adolescents’ psychological distress increased over the course of the pandemic.

## 1. Introduction

In March 2020, COVID-19 was declared a pandemic, and since, children’s and adolescents’ lives were turned upside down by lockdown measures such as school closures. Negative consequences of lockdown procedures on mental health were repeatedly demonstrated [1,2]. At the same time, sleep research has pointed out the special case of adolescents: Early morning school start times conflict with their late sleep biology, and thus adolescents typically do not get enough sleep on school days with negative impacts on health and well-being [3,4,5,6,7]. Hence, pandemic high school closures allowed adolescents to get up later in the morning, resulting in longer sleep duration [8,9,10,11,12]. Recently, we demonstrated two opposing effects on adolescents’ health and well-being during school closures in Switzerland by comparing a control group collected in spring 2017 and a closure group collected in spring 2020 when schools were closed: The closure group showed longer sleep duration on school days, better health-related quality of life (HRQoL), and less substance use than the control group [13]. Additionally, sleep duration on school days was associated with better HRQoL and less caffeine use, but, concurrently, depressive symptoms related inversely to the same variables. Thus, a beneficial effect coexisted with a detrimental effect of psychological distress. Our interpretation was that beneficial effects resulted from later wake times due to homeschooling and that they should thus be specific to pandemic phases of school closures. On the contrary, psychological distress was assumed to be a general pandemic effect (i.e., unspecific to schools open vs. closed). However, the design did not allow us to differentiate between general pandemic effects and impacts specific to school closures. Moreover, cohort effects could not be excluded. Therefore, to investigate how sleep behavior and health-related characteristics developed in the course of the pandemic when schools were open again, we assessed another sample in spring 2021 still under pandemic conditions, but with no school closures in place (postclosure group). We hypothesized that the postclosure group would still show signs of psychological distress as we previously found in the closure group, but that sleep behavior and health-related characteristics would be similar again as in the pre-pandemic control group.

## 2. Materials and Methods

### 2.1. Participants

Students from 21 high schools in the Canton of Zurich, Switzerland, were invited to participate anonymously in online surveys at three different time points: In spring 2017 (pre-pandemic control group, *n* = 5308) [13,14], in spring 2020 during pandemic high school closure (closure group, *n* = 3664) [13], and in spring 2021 still under pandemic conditions, but when schools were open (postclosure group, *n* = 3266). Surveys were identical except that in 2017, we additionally asked students about school start time preferences [14]. In 2020 and 2021 (pandemic groups), we additionally assessed depressive symptoms and COVID-19 related variables (see below for details). In 2017, 17 of 20 schools participated in the survey study by circulating the survey link among students. The median participation rate was estimated as 38% (interquartile range (IQR) 35–43). In 2020 (closure sample), 21 of 21 schools participated in the survey with a median participation rate of 23% (5–42). Lastly, in 2021 (postclosure sample), 19 of 21 schools participated with a median participation rate of 24% (17–39). Students were included in the analysis if they provided sex, age, and school, and did not currently suffer from COVID-19. For the postclosure group, students in homeschooling were excluded. Ethical approval and informed consent were not required because all surveys were anonymous as confirmed by the local ethics committee.

### 2.2. Measures

**Sample characteristics**: Students provided their sex, age, and mother tongue (Swiss German vs. other), and indicated which school they attended and whether they suffered from a physical disease or mental illness (yes/no).

**Sleep characteristics**: Sleep-wake patterns on school (scheduled) days and weekend (free) days were assessed using the Munich Chronotype Questionnaire (MCTQ) [15]. Sleep period on school and weekend days was calculated as the time between bedtime and wake-up time. Additionally, students indicated on how many nights in the past two weeks they had had difficulties falling asleep or sleeping through.

**Health-related characteristics**: Health-related quality of life (HRQoL) was assessed using the KIDSCREEN-10 questionnaire [16]. Adolescents indicated their weekly caffeine consumption separately for four caffeine-containing beverages (coffee, energy drinks, Coca-Cola-like sweet drinks, and black, white, or green tea) on a scale from never (0) to every day (5). Answers to the four items were summed up, leading to a total score of 0–20 (higher scores indicate greater caffeine consumption). Students older than 16 years were asked analogously about their weekly alcohol consumption of beer, wine, and spirits (total score: 0–15, higher scores indicate greater alcohol consumption). Additionally, they indicated whether they smoked and if so, how many cigarettes per weekday and on a typical weekend. Daily cigarette consumption was then calculated as the weighted mean (five times cigarettes per day plus weekend divided by seven). In the closure and postclosure groups only, depressive symptoms were assessed with the withdrawn/depressed scale of the Youth Self Report (YSR/11-18R) [17]. Raw scores were transformed to T values (normative values with a mean of 50 and a standard deviation of 10). T values of 70 or higher represent clinically relevant levels of depressive symptoms [17].

**COVID-19-related measures**: The closure and postclosure groups indicated how much their social contacts, use of digital media, and sports changed since the pandemic and how much they worried that the pandemic would affect their future academic achievement (1: not at all–5: very strongly).

### 2.3. Statistical Analyses

Sample characteristics were compared using analysis of variance or chi^2^-tests, and *p* values smaller than 0.05 were considered significant. Mixed models (lmerTest package [18]) were used to investigate between-sample differences in sleep and health-related characteristics with the control sample as the reference category (participation in the respective sample was added to the model, 0 = control sample). All models additionally included sample characteristics as control variables (sex, age, mother tongue, physical disease, mental illness) and school as a random effect, and results were corrected for multiple comparison (Bonferroni method). Depressive symptoms and changes since the pandemic only assessed in the lockdown and pandemic samples were directly compared between them (closure sample as reference category). Semi-partial *R*^2^_β*_ (r2glmm package [19]) were used as effect sizes of fixed effects.

## 3. Results

### 3.1. Sample Characteristics

In total, survey responses of 12,238 high school students were investigated. The three samples differed significantly in age (control: median 16, inter-quartile range (IQR) 15–17; closure: 16, 15–17; postclosure: 16, 14–17, *p* < 0.001), mother tongue (67.7% Swiss German native speaking vs. 65.1% vs. 63.2%, *p* < 0.001), presence of physical disease (7.6% vs. 5.3% vs. 7.3%, *p* < 0.001), and presence of mental illness (5.2% vs. 5.0% vs. 8.7%, *p* < 0.001). Sex distribution tended to be different across samples (65.1% female vs. 66.3% vs. 67.5%, *p* = 0.06). Therefore, all these sample characteristics were included as control variables in the mixed models.

### 3.2. COVID-19-Related Measures

Perceived changes in social contacts, use of digital media, sports, and worries about future academic achievement due to the pandemic were significantly less pronounced in the postclosure than in the closure sample (all *p* values < 0.01). Strong or very strong changes were reported by approximately half of the closure and more than a third of the postclosure group (social contacts: by 54.8% and 36.8% of participants; digital media: 52.8% and 40.8%; sports: 50.9% and 35.5%). To worry strongly or very strongly about future academic achievement was indicated by 32.2% of the closure and 25.1% of the postclosure group.

### 3.3. Sleep Behavior

**Figure 1:**
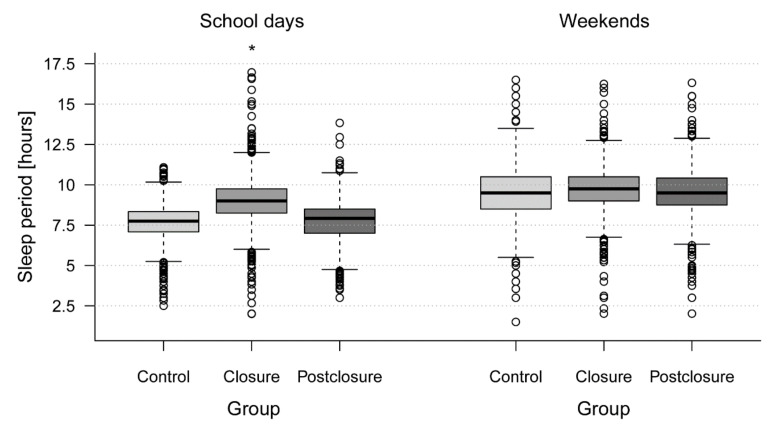
Sleep period on school days and weekends across all three groups (control group assessed under regular conditions in Spring 2017, closure group assessed during pandemic high school closures in Spring 2020, postclosure group assessed under pandemic conditions but schools were open in Spring 2021). * Mixed models were used to investigate differences between the control group (reference category) and the pandemic samples (closure and postclosure group) while controlling for sociodemographic variables and school (random effect), separately for sleep period on school days and on weekends. Sleep period on school days was significantly longer in the closure compared to the control group (*p* < 0.001). No significant difference between the postclosure and control group’s sleep period on school days was found and the groups showed similar sleep periods on weekends.

Results of between-sample comparisons can be found in Table 1 and Figure 1. On school days, the closure groups’ sleep period (median 9, IQR 8.25–9.75) was on average 75 min longer compared to the control group (7.75, 7.08–8.33). The postclosure group showed comparable values to the control group (7.92, 7.00–8.50). On weekends, the three groups showed similar sleep-wake patterns.

Difficulties falling asleep and problems sleeping through the night were significantly more frequently reported in both the closure (33.8% and 11.9% more than four times in the past two weeks) and the postclosure group (37.3% and 13.4%) compared to the control group (30.9% and 8.3%; Table 1).

### 3.4. Health-Related and Behavioral Characteristics

HRQoL was significantly higher in the closure group (median 44.48, IQR 40.24–49.76) and lower in the postclosure group (42.27, 36.51–48.29) compared to the control group (42.27, 37.42–48.29; Table 1). Adolescents in the closure group consumed significantly less caffeine and alcohol than the control group, while the postclosure and control groups showed similar values. Nicotine consumption was comparable across all three samples.

Depressive symptoms were only assessed in the closure and postclosure groups and were thus compared directly between them: The postclosure group (median 58, IQR 54–69, 16.6% ≥ 70) reported significantly higher symptom levels than the closure group (57, 51–64, 9.1% ≥ 70; *n* = 5592, B (SE) = 2.95 (0.49), *p* < 0.001, *R*^2^_β*_ = 0.02 [95% confidence interval: 0.01, 0.03]).

## 4. Discussion

During pandemic high school closures, adolescents slept over one hour longer on school days and showed better HRQoL and less substance use [13]. These beneficial effects were no longer observed one year later in the course of the pandemic when schools were open again. This might be because students could not benefit from homeschooling anymore and had to get up similarly early. On the contrary, the postclosure group showed evidence for a chronic manifestation of psychological distress in the course of the pandemic as indicated by higher levels of depressive symptoms, worse HRQoL, higher prevalence of diagnosed mental illness, and more sleep problems.

In our previous publication [13], we described two opposing associations of homeschooling with adolescents’ health and well-being during pandemic school closures: On the one hand, there was a beneficial effect of longer sleep duration on school days, reflected in the association between longer sleep duration and better HRQoL as well as less caffeine use. On the other hand, however, depressive symptoms were inversely related with the same variables (i.e., worse HRQoL and higher caffeine use), which was interpreted as pandemic-induced psychological distress not specific to school closures. However, this interpretation remained speculative. The new findings presented in this paper allow more precise characterization: As expected, better HRQoL and less substance use were specific to the closure group and not observed anymore in the postclosure group, further supporting that better health-related characteristics were related to homeschooling that allowed later wake times and thereby longer sleep duration. In contrast, as found in other studies, psychological distress seems to apply to the pandemic in general and even intensified with increasing time since the outbreak [20]. Although the postclosure group showed signs of habituation to the pandemic (less perceived changes in different areas of life (i.e., social contacts, sports, digital media) and less worry about future academic achievement since the pandemic), depressive symptoms were significantly higher than in the closure group assessed one year before (16.6% vs. 9.1% indicated clinically relevant levels) [1,2,21].

Additionally, to the intrinsic limitations of anonymous and pseudo-longitudinal online surveys described elsewhere in detail [13], it has to be considered that pandemic measures did not only differ in schools closed vs. open, but measures implemented in spring 2021 were generally less strict than in spring 2020. Furthermore, also other aspects not assessed in our surveys such as family characteristics or technical equipment at home might have influenced sleep and health-related characteristics during the pandemic lockdown.

## 5. Conclusions

In conclusion, while the closure group showed a net positive figure in sleep and health compared to the pre-pandemic control group, these beneficial effects were not found anymore in the postclosure group that no longer had the opportunity for more sleep due to school closures. Therefore, delayed morning school start times for adolescents might serve to counteract the negative consequences of the pandemic.

## Figures and Tables

**Table 1:** Regression coefficients of the sample main effects in models with different dependent variables.

		Closure vs. Control Sample			Postclosure vs. Control Sample		
Dependent Variable	*n*	β (SE)	*p* Value (Uncorr.) ^a^	*R*^2^_β*_^b^ [95% CI]	β (SE)	*p* Value (Uncorr.) ^a^	*R*^2^_β*_^b^ [95% CI]
**Sleep characteristics**							
School days: bedtime	9861	0.35 (0.03)	<0.001 (<0.001)	0.02 [0.02, 0.03]	0.09 (0.03)	0.01 (<0.001)	0.001 [0.00, 0.003]
School days: wake-up time	9777	1.50 (0.09)	<0.001 (<0.001)	0.42 [0.40, 0.43]	0.08 (0.03)	0.32 (0.03)	0.002 [0.00, 0.004]
School days: sleep period	9777	1.18 (0.08)	<0.001 (<0.001)	0.17 [0.16, 0.19]	−0.05 (0.04)	>0.99 (0.21)	0.00 [0.00, 0.001]
Weekends: bedtime	9850	−0.10 (0.07)	>0.99 (0.20)	0.001 [0.00, 0.002]	0.03 (0.05)	>0.99 (0.62)	0.00 [0.00, 0.001]
Weekends: wake-up time	9819	0.06 (0.05)	>0.99 (0.22)	0.00 [0.00, 0.002]	−0.06 (0.05)	>0.99 (0.25)	0.00 [0.00, 0.001]
Weekends: sleep period	9818	0.16 (0.05)	0.07 (0.01)	0.003 [0.001, 0.01]	−0.06 (0.04)	>0.99 (0.20)	0.00 [0.00, 0.001]
Difficulties falling asleep ^c^	9869	0.16 (0.03)	<0.001 (<0.001)	0.004 [0.002, 0.01]	0.26 (0.03)	<0.001 (<0.001)	0.01 [0.01, 0.01]
Problems sleeping through ^c^	9869	0.15 (0.02)	<0.001 (<0.001)	0.004 [0.002, 0.01]	0.26 (0.03)	<0.001 (<0.001)	0.01 [0.01, 0.02]
**Health-related characteristics**							
HRQoL	9819	1.51 (0.30)	0.002 (<0.001)	0.01 [0.003, 0.01]	−1.23 (0.31)	0.02 (0.002)	0.004 [0.002, 0.01]
Smoking (# cigarettes/day) ^d^	402	−1.38 (0.59)	0.24 (0.02)	0.01 [0.00, 0.05]	−1.28 (0.57)	0.29 (0.02)	0.01 [0.00, 0.04]
Alcohol (score 1–15) ^e^	5998	−0.49 (0.07)	<0.001 (<0.001)	0.01 [0.01, 0.02]	−0.13 (0.07)	0.90 (0.07)	0.001 [0.00, 0.002]
Caffeine (score 1–20)	9869	−0.63 (0.08)	<0.001 (<0.001)	0.01 [0.004, 0.01]	−0.06 (0.08)	>0.99 (0.44)	0.00 [0.00, 0.001]

Notes: In all models, age, sex, primary language, physical disease, and mental illness were also included as fixed effects, and school was included as a random effect. CI = confidence interval, HRQoL = health-related quality of life, SE = standard error, uncorr. = uncorrected ^a^ *p*-values corrected for multiple comparisons (Bonferroni method: uncorrected *p* value multiplied by 12). ^b^ Semi-partial *R*^2^ statistic [19]. ^c^ Square-root transformed. ^d^ Only students older than 16 years who smoked were included ^e^ Only students older than 16 years were included.

## Data Availability

The data presented in this study are available on request from the corresponding author. The data are not publicly available due to the agreement with participating schools to only share data with third parties on request.
